# The Rise and Demise of Integrated Pest Management in Rice in Indonesia

**DOI:** 10.3390/insects6020381

**Published:** 2015-04-17

**Authors:** Craig Thorburn

**Affiliations:** Centre for Geography and Environmental Science, Monash University, Victoria 3800, Australia; E-Mail: Craig.Thorburn@monash.edu; Tel.: +61-3-9905-9319; Fax: +61-3-9905-2948

**Keywords:** integrated pest management (IPM), rice, rice brown planthopper (BPH), *nilaparvata lugens* stål, insecticide-induced resurgence, Indonesia

## Abstract

Indonesia’s 11-year (1989–1999) National Integrated Pest Management Program was a spectacularly successful example of wide-scale adoption of integrated pest management (IPM) principles and practice in a developing country. This program introduced the innovative Farmer Field School model of agro-ecosystem-based experiential learning, subsequently adapted to different crops and agricultural systems in countries throughout the world. Since the termination of the program in 1999, Indonesia has undergone profound changes as the country enters a new era of democratic reform. Government support for the national IPM program has wavered during this period, and pesticide producers and traders have taken advantage of the policy vacuum to mount an aggressive marketing campaign in the countryside. These factors have contributed to a reappearance of the pesticide-induced resurgent pest problems that led to the establishment of the National IPM Program in the first place.

## 1. Introduction

Rice is one of the most important human food crops, occupying one-tenth of the world’s arable land and accounting for around 20 per cent of total global grain production. In some Asian countries rice comprises up to one third of total planted area. Since World War II, the total global area planted in rice has increased by 67 percent, yields have increased by 95 percent, and total world production trebled [1]. Compared to other grains, however, a relatively small proportion of the world’s rice crop is traded internationally [2]. Therefore, self-sufficiency in rice is perceived as key to ensuring national food security in countries where rice is the main staple [3].

Indonesia, like most other South and Southeast Asian countries, undertook an aggressive rice intensification program from the 1960s onward, employing Green Revolution “packages” of high-yielding varieties (HYV), chemical fertilizers, insecticides, improved irrigation and agricultural extension [4,5]. During the 1970s and ‘80s, Indonesia became one of the greatest success stories of this approach, increasing average rice yields from two to four and a half tons per hectare (the highest in tropical Asia); transforming from being the world’s largest rice importer in the 1960s, to achieving self-sufficiency in rice by 1984 [6,7].

However, dependency on a small number of HYV varieties combined with prophylactic application of chemical pesticides rendered the country’s rice farmers vulnerable to plagues of insecticide-induced resurgent pests, particularly the rice brown planthopper (BPH, *Nilaparvata lugens* Stål), which threatened to undermine the impressive production gains of the Green Revolution varieties and techniques.

Beginning in the mid-1980s, Indonesia introduced integrated pest management (IPM) as a new crop management approach, developing the Farmer Field School (FFS) model of empowering farmers to make informed crop management decisions. Both IPM and the FFS model represented radical departures from the agricultural intensification and extension models that preceded them, shifting from the top-down dissemination of knowledge, instructions and inputs, to a farmer-driven crop management strategy based on ecosystem health and the simple principle of “grow a healthy crop”. The program achieved prodigious results during its 14-year duration, and helped to fundamentally transform the relationship between Indonesia’s agricultural bureaucracy and the country’s millions of rice farmers.

However, during the political and social upheaval leading up to and following the resignation of Indonesia’s strongman president Suharto in 1998, national and local government support for IPM principals and programs in Indonesia has wavered. Today, a decade and a half into Indonesia’s democratic *reformasi* era, rice farmers are using more pesticides than ever, with the consequence that the country is again experiencing devastating BPH infestations in many of its key rice-producing areas.

This article relates the story of the successful promotion of IPM in rice in Indonesia, followed by a discussion of the national agriculture bureaucracy’s retreat from IPM approaches and concomitant resurgence of the BPH pest. The agroecology of rice itself plays a central part in this story, as does the role of Indonesia’s rice farmers. Before discussing the introduction of IPM in rice in Indonesia, it is useful to briefly review the history of the development and spread of HYV rice varieties, as well as Indonesia’s experiences with Green Revolution technologies.

The article draws on several years’ observation and conversations with key protagonists of Indonesia’s national IPM program. The author lived and worked in Indonesia throughout most of the period described in the article. Although he never worked directly for the program, his work with Indonesian and international NGOs and bilateral aid projects put him in close contact with many key players. Beginning in 2006, several years after the termination of the national IPM program in 1999, he was encouraged by colleagues who formerly worked with the program—many of whom were still engaged in the promotion of sustainable agriculture and farmer empowerment in Indonesia—to write the story of the national IPM program, in particular the evolution of the Farmer Field School approach. In 2006 and again in 2012, the author spent several weeks traversing the island of Java meeting with IPM farmer group members, activists, academics, and pest control workers from the Ministry of Agriculture and regional agricultural agencies, to attempt to assess the resilience and sustainability of the IPM program’s message, approaches and institutional infrastructure. This article is the result of these explorations.

A caveat is in order regarding statistical and technical data used in—or absent from—this report. Accurate statistical data is notoriously difficult to come by in Indonesia. Many factors contribute to this situation, including the generally poor quality of government data [8], the perceived political sensitivity of rice production statistics and information on rice pest infestations and crop losses, and a legacy of suspicion and mistrust among Indonesian government bureaucracies toward foreign researchers [9]. Some fairly accurate macro-level data were available from reports produced by or for the National IPM Program during the years of its existence. Descriptions and analysis of the more recent increase in insecticide use and concomitant BPH resurgence draw from direct observations, conversations with Indonesian farmers, academics, students and a few government officials and fieldworkers. Studies of this phenomenon undertaken by Indonesian academics rely entirely on small-scale field surveys in a smattering of affected areas. To date, the results of these studies have only been published on researchers’ websites and some Indonesian newspapers and magazines.

## 2. The (Human) Ecology of Rice

Rice has fed more people over a longer period than has any other crop, and is presently the source of one quarter of global per capita caloric intake [10]*.* Chang [11] (p. 143) postulates that the cultivation of Asian rice began in “a broad belt that extended from the Ganges plains below the foothills of the Himalayas, across Upper Burma, northern Thailand, and Laos, to North Vietnam and south China between 10,000 and 4000 BC”.

In the early Neolithic era, rice was cultivated in clearings in the forests, direct seeded and dependent on rainfall for water. The techniques of puddling the soil and transplanting seedlings probably developed independently in several locations in China as early as the 2nd or 3rd millennium BC [10]. These innovations, along with the discovery of early maturing and photoperiod insensitive varieties, led to rice becoming the most highly productive grain crop that came to dominate agricultural production throughout Asia [12]. Migrants from the Asian mainland brought wetland rice cultivation to the Philippines during the second millennium BC, and Deutero-Malays carried the practice to the Indonesian archipelago in about 1500 BC.

Wet-land rice cultivation is much more demanding than other staples such as wheat, barley or millet, both in terms of labor and also in human ingenuity, inventiveness and organization. It also supports high concentrations of human population [13]. The Indonesian island of Java, for instance, comprises less than seven per cent of the country’s total land area, but produces over 53 per cent of its rice, and is home to 57 per cent of the country’s population [14,15].

The extensive geographical distribution of wet-land rice cultivation, combined with its long ecological history, has resulted in the creation of a vast man-made ecozone. Despite being mainly a monocrop system, the ecological complexity of wet-land rice is unique among the world’s agro-ecosystems. The arthropod species richness of tropical rice fields surpasses that of most natural (as opposed to man-made) temperate systems [16]. The rice field agro-ecosystem is particularly stable as a result of an extremely rich web of generalist predators. An early-season abundance of detritus- and plankton-feeding insects provides a well-dispersed food source for a diverse community of generalist predators, allowing predator populations to develop well in advance of rice-pest populations. Pest infestations, therefore, usually remain well below economically damaging levels [17]. While numerous host-specific parasites and parasitoids play crucial roles in these complex webs, it is the early season prevalence of generalist predators—spiders, water striders, dragonfly and damselfly nymphs and adults, and ground beetles—that makes the wet-rice agro-ecosystem so uniquely stable and robust [18,19].

These in-field ecological webs can be enhanced or disrupted by many different habitat factors—*i.e.*, spatial planting patterns, staggered or synchronous planting, fallow periods or inter-cropping, water-use patterns, the organic content of paddy soil and water, whether stubble is burned or left to decay, and the use of insecticides and other agricultural chemicals. Variations in any of these factors affect predator and prey communities living on the plants, on the water surface, and in the water [16].

Countless generations of intense interaction with these systems has produced a wealth of place-specific farmer’s knowledge of biological and cultural controls. Much of this knowledge has been discounted by the promoters of agricultural modernization. One of the prominent features of the science-based Green Revolution in rice has been standardization—of varieties, and of crop management knowledge and practice. To date, most scientific research relating to arthropods in tropical rice (and management strategies produced) has been directed at only a small handful of “pest” species, without examining the biotic linkages to the rest of the system [16].

## 3. The Green Revolution in Rice

The term “Green Revolution” was first coined by William Gaud, Director of USAID, in a speech to the Society for International Development in Washington, DC. He said:

*These and other developments in the field of agriculture contain the makings of a new revolution. It is not a violent Red Revolution like that of the Soviets, nor is it a White Revolution like that of the Shah of Iran. I call it the Green Revolution*.

[20]

A highly emotive term, the “revolution” in cereal production in developing countries is alternately credited with preventing a billion famine deaths [21], or causing a host of dire social and environmental problems that threaten the livelihoods of billions more [22,23].

Regardless of ideological standpoint, few would dispute Dalrymple’s [24] (p. 1067) claim that “high-yielding varieties (HYVs) … of wheat and rice have spread more widely, more quickly, than any other technological innovation in the history of agriculture”. Since the International Rice Research Institute (IRRI) released its first successful semi-dwarf variety IR 8 in 1966, the total area planted in HYV rice in Asia, Latin America, the Near East and Africa has increased yearly [1,10]. In Indonesia, more than 85 per cent of wet-rice *sawah* is planted in High-Yielding Varieties [25]. Combined with improved irrigation, HYVs seeds have been the most important factor in the country’s spectacular threefold increase in rice production [5,7].

Spearheaded by the Rockefeller Foundation and USAID-supported Consultative Group on International Agricultural Research (CGIAR), the Green Revolution epitomizes the cold-war-era approach to rural development through technological, rather than social, engineering. The basic goal of CGIAR cereal research and dissemination efforts was to develop crop varieties and improved cultural practices that could increase production at a rate that exceeded population growth in developing countries. To achieve the necessary yield increases, the approach depends on intensive cropping, synchronized maturation, heightened reliance on mechanization, precise water control, and intense and precisely timed chemical inputs [26].

At the core of Green Revolution package stand the short, stout stalks of semi-dwarf HYVs. Early efforts to improve yields ran into problems when increased grain weight caused plants to lodge (bend or topple over) [24]. To address this problem, plant breeders began searching for short varieties of wheat and rice to cross with their high-producing strands. Other desirable characteristics were early maturity and photo-insensitivity, which would support multiple cropping. The first breakthrough in rice came from crossing a semi-dwarf variety from Taiwan, *Dee-geo-woo-den* (DGWG), with a tall, vigorous Dutch strain developed in Java in the 1940s, known as *Peta*. The marriage of these two remarkably different rice varieties launched the Green Revolution in rice in South and South-East Asia [27]. The resulting cross, known officially as IR-8-288-3 and popularly as IR 8, tillered profusely in response to applications of nitrogen, producing numerous heavy panicles of grain. Its thick culms and great straw strength prevented lodging. In field trials at IRRI, IR 8 produced yields of up to 10.13 tons of paddy per hectare [4].

IR 8 was released for distribution in 1966, and rapidly adopted throughout Asia. Its release was quickly followed by several new varieties developed by IRRI, the University of the Philippines, and the Indonesian government’s own rice-breeding program. Throughout the 1970s, the area planted in IRRI and IRRI-derived high-yielding varieties steadily increased, reaching 67 per cent of rice planted in Indonesia by the end of the decade [5].

To successfully grow high-yielding varieties, farmers are required to carefully control water, necessitating major national investments in irrigation infrastructure. The timing and volumes of chemical fertilizer and other inputs are crucial, again requiring major government investments in production, storage and distribution facilities for seed, fertilizer and chemical pesticides. As well, a national agricultural extension program was needed to instruct and inform farmers in the new methods and techniques. The programs were often accompanied by provision of credit to enable farmers to purchase the necessary inputs [5,7].

By the mid-1970s, when the “revolution” had been underway for a decade, IRRI scientists were becoming concerned that the loss of genetic diversity was creating vulnerability to pest and disease outbreaks. Virtually all semi-dwarf varieties carried the same dwarfing gene from DGWG, while three quarters of the Indonesian HYVs could be traced to the same maternal parent, *Cina* (one of the component varieties of the Dutch crossbreed *Peta*) [27].

The first problems began to appear at IRRI just a first few years into the breeding program, when test plots began evincing BPH infestations and symptoms of hopperburn (see below). Rice brown planthopper is a common insect in rice fields throughout Asia, but had never caused major problems in tropical rice prior to the development and spread of Green Revolution approaches. With the advent of HYV seeds and technological packages, this began to change. By the mid-1970s, BPH outbreaks were beginning to cause crop losses in Indonesia and other Asian countries that had adopted the new varieties [28]. At IRRI, research priorities shifted to developing and disseminating new BPH-resistant rice varieties—an approach that worked for a while [16]. However, within a few seasons, new BPH biotypes appeared that could feed on the resistant varieties, signaling that new approaches would be needed [4].

As previously mentioned, during the 1970s and ‘80s Indonesia achieved the greatest success of any country in adopting and disseminating HYV rice varieties. As associated problems began to surface, therefore, Indonesia was also the most vulnerable of any country. The following section describes Indonesia’s rice intensification program from its outset in the early 1960s, through attainment of rice self-sufficiency in 1984, until its near-unravelling only two years later due to major BPH outbreaks in the “rice bowls” of northern Java.

## 4. The BIMAS Mass Guidance Rice Intensification Program

BIMAS (for *Bimbingan Massal*, or Mass Guidance) was the name given to Indonesia’s national rice intensification program, as well as the special coordinating body and governmental network established to direct it. Over the years, it spawned a number of sub-programs, with acronyms such as INMAS (Mass Intensification), INSUS (Special Intensification) and SUPRA-INSUS (which required groups of 50 to 100 farmers with contiguous fields to make joint decisions). As well, the BIMAS acronym and approach has also been adapted to other crops and commodities besides rice. The following section focuses on the original, rice intensification BIMAS program.

Although the BIMAS program was initiated two years before the military coup that brought President Suharto’s New Order government to power, its title encapsulates two of the most fundamental precepts of the new regime’s character, “mass” and “guidance”. *Massal*, the second half of the BIMAS acronym, conjures the notorious “Floating Mass” policy introduced by Indonesia’s military leaders in the wake of the 1965 coup and genocide, which prohibited any organized political activity in villages or rural areas. The notion underlying the Floating Mass was that Indonesia’s un-sophisticated multitudes must not be distracted from the task of development by politics [29]. According to General Ali Moertopo, one of the main architects of New Order socio-political policy, prior to the 1965 coup, the Indonesian people—“who consist mostly of honest, good-willed and simple peasants”—had been led into “disorder and fanaticism” by “fall[ing] prey to the political and ideological interests of [political] parties”. Politics, therefore, was seen as a dangerous and divisive factor, best left to trusted and competent leaders who could guide the political process to “support the realization of development in all fields of human life in order to increase the people’s welfare’ [30] (pp. 83–84).

If it was politics that nearly tore the republic apart, Indonesia’s new leaders were convinced that development would stitch it back together again. Development became the new national project. It was the job of politicians and the state bureaucracy to guide the Indonesian people toward this all-encompassing goal [31]. The country’s millions of microfundia rice farmers became pliable atoms in a floating mass, needful of strong guidance if they were to perform their important function of feeding the nation.

To guide and manage the development process, the New Order government assembled a vast national bureaucracy extending to the furthest reaches of the archipelago, down to the village level. The hierarchical structure of central government, provinces, districts, sub-districts and villages, mirrored the Indonesian military’s territorial command structure. The BIMAS program, like all other national development programs, was structured along these lines. The program delivered credit, agricultural inputs, and guidance conceived by the center and filtered down through layers of territorial structures.

“*Bimbingan*”—the other half of the BIMAS acronym—means “leadership” or “guidance” in Indonesian. Under the BIMAS program, Indonesia’s rice farmers were led and guided to deliver the huge increases in rice production needed to feed the growing nation. The initial target was to reach self-sufficiency in rice by the end of the first (1968–1973) Five Year Development Plan [32].

BIMAS was, at its core, a farm credit program, but the name came to encompass the entire range of Green Revolution packages of improved seeds, chemical fertilizer, improved irrigation, logistical support, and agricultural extension that fuelled the rice self-sufficiency endeavor. BIMAS became the emblematic program of the New Order government—and surely one of its most successful [7].

BIMAS exemplified the regime’s technocratic nature. The introductory paragraph of first article on the new program to appear internationally effuses:

*The BIMAS programmer of agricultural extension was conceived by a small group of young men without prior experience in extension work. The people who work with the farmers are also young and without extension experience, fourth and fifth-year students from the colleges of agriculture. Yet the programmer has been very successful in encouraging farmers to increase their production*.

[33] (p. 60).

The program experienced the usual problems during its initial years, including the failure of cooperatives in many BIMAS areas; inadequacies of credit supply and delays in delivery; delayed delivery of agricultural inputs; and “partial failure of communication to the farmers as to the real benefits and how to realize them by use of particular quantities of nitrogen and phosphate fertilizers and related pesticides” [32] (p. 37). By 1968, the program had managed to stem the decline in Java’s rice production, but was still unable to produce an increase in production needed to match or exceed population growth on the island [7].

Many of the initial problems were resolved by the mid-1970s, and credit disbursements increased by a factor of seven between 1971 and 1976, from Rp. 8.5 billion to 55.1 billion. Repayment rates were abysmal, with BIMAS arrears reaching as high as 64 per cent of total loans outstanding in the late 1970s [34]. The program did, however, succeed in disseminating HYV seeds and accompanying agricultural inputs and instructions to large numbers of Indonesian rice farmers. As the BIMAS program’s early problems were overcome, Indonesia’s rice intensification program grew to become the most successful in the world.

With the OPEC price increases of the late 1970s, the Indonesian government was flush with oil revenues, and thus able to aggressively pursue its rice intensification agenda. Various authors ascribe different facets of the BIMAS program with primary causation for the country’s impressive increases in rice production. Fox, for instance, points to the massive investments in irrigation infrastructure and HYV seed, chemical fertilizer and pesticide production and distribution [4,5], while most economists argue that price policy was the primary driving force [7,34,35].

As previously mentioned, Indonesia did reach its goal of self-sufficiency in rice in 1984, some fifteen years after launching the national rice intensification program. In recognition of this achievement, the Director General of the United Nations Food and Agriculture Organization (FAO) presented President Suharto a gold medal bearing his image at the organization’s biennial meeting in November 1985 [36]. At home, Suharto had taken on the title of “*Bapak Pembangunan*”—the “Father of Development” [31].

### 4.1. Train and Visit, Spray and Pray

Central to the success of the BIMAS program was the tremendous effort put into developing a national agricultural extension infrastructure, to deliver information and instructions to farmers. Indonesia was a primary site of development of the Training and Visit (T&V) agricultural extension methodology, later promoted by The World Bank in over 70 countries worldwide [37].

Indonesia’s New Order government enjoyed a special relationship with the World Bank [38]. Indeed, according to a recent history of the World Bank, “Indonesia was the presidentially designated crown jewel in the Bank’s operational crown” [39] (p. 493). Surely, Indonesia’s agricultural intensification program was one of the most sparkling gems in the array. According to World Bank annual reports from the years 1976 to 1987, the Bank loaned Indonesia $134 million to support T&V extension services, roughly five per cent of the $3 billion invested in rice intensification during that period (the majority being for irrigation ($2.1 billion) and nitrogen fertilizer production and distribution ($460 million)).

The T&V system stressed exclusive dedication to technical information dissemination through a single hierarchical line of command; in-house technical specialists to provide training to staff and tackle issues reported by field staff; a strict and predetermined schedule of village visits over a two-week cycle to disseminate the key set of messages for the coming two-week cycle [40]. By 1984, at the height of the BIMAS program in Indonesia, some 14,000 fieldworkers paid fortnightly visits to groups of 16 “contact farmers” who were in turn responsible to spread the BIMAS message another 160 to 320 other farmers [6]. These extension workers were also responsible for organizing the distribution of BIMAS packages of seeds and chemicals. By the mid-1980s, over half the harvested area of *Padi sawah* (irrigated rice) in Indonesia was under the BIMAS program.

The system included means of exerting “gentle pressure”, if not outright coercion, on farmers in order to meet targets for the use of improved seeds and other inputs. Agricultural inputs were distributed through the village administration. Farmers purchasing seed on credit through the Village Cooperative (KUD) were required to accept the entire package of seeds, fertilizers and pesticides prescribed as part of the blanket recommendations covering the entire rice-producing area. In some areas, crops of farmers who were not planting the latest HYVs were cut down by village officials, or planting of HYVs and fertilizer use were enforced by the army [6].

The cultivation of HYVs over an extensive area increases the new rice’s susceptibility to pest infestations. High fertilizer use, close spacing, the dense canopy produced by heavy tillering, creates a favorable situation for the multiplication of certain kinds of pest [41]. The BIMAS approach mandated calendar-based applications of insecticides as the primary means of addressing potential pest problems [16].

Chemical pesticides are called “*obat*” in Indonesian, which means “medicine”. The implication being that these various types of “medicine” will help the rice plant stay healthy, in the same way that immunizations protect humans [42]. As with urea and superphosphate fertilizers, the Indonesian government heavily subsidized agricultural pesticides. National expenditures on pesticide subsidies rose from US$50 million per year in the 1970s to over $150 million per year in the mid-1980s [16]. For a period from the late 1960s to the mid-1970s, the government contracted with various international insecticide firms to conduct aerial spraying of large areas of rice land, in an attempt to curb stem-borer infestations [16].

A growing body of scientific research indicates that, while doubling or trebling nitrogen fertilizer application leads to significantly increased production of HYV rice, there is no parallel evidence to show that increasing insecticide use leads to measurable increases in yields (nor, conversely, that reduced pesticide use results in reduced rice yields) [43,44]. In spite of this, 12 per cent of pesticides sold worldwide are applied to rice crops, more than any other crop [45]. Approximately 80 per cent of this amount used in Asia [46].

This combination of factors, including aggressive promotion of HYV rice varieties as part of a Green Revolution package that included heavy-handed extension by trained experts in conjunction with local officials and the military, massive government subsidies on chemical fertilizers and pesticides, and prophylactic use of insecticides to control rice pests, together created a perfect storm for major outbreaks of insecticide-induced resurgent pest outbreaks, specifically, for a rice brown planthopper plague of unprecedented proportions.

### 4.2. BPH: An Archetypical Insecticide-Induced Resurgent Pest

The rice brown planthopper, *Nilaparvata lugens* Stål (Hemiptera: Delphacidae)—henceforth BPH—is native to all rice-growing areas of Asia, but was hardly heeded until the height of the Green Revolution, when it suddenly became a pest. The two major international texts on Asian rice pests published in the 1960s [47,48] do not even contain pictures of this insect.

The BPH is a small (2–3.5 mm in body length), brownish, sucking insect, belonging to the suborder Delphacidea. Like other homopterous insects, it has a set of four stylets in its mouth which serve as a piercing and sucking organ [49]. The BPH is a typical vascular feeder; it primarily sucks the phloem sap from fresh rice plants. Female adults of BPH spend about 60%–90% of their time sucking on rice plants, with repeated sustained sucking lasting one to seven hours. In addition to removing photosynthates from the plant, the sucking can block the vascular bundles in the rice leaves, causing toxic build-ups of free amino acids, leading to a phenomenon called “hopperburn”. Hopperburn appears initially as a yellowing of older leaves, but soon extends to the whole plant, which turns brown and dies. Non-fatal infestations generally result in fewer panicles per plant, fewer grains per panicle, and lower percentages of ripened grain and lighter grain weight, depending on what stage of the rice plant’s growth the infestation occurred [50].

Female BPH embed their eggs in the leaf sheaths of rice, which thereby escape insecticide sprays [1]. The lifecycle of the BPH is a mere 22 days, and a single female lays 200 to 400 eggs. In unsprayed wet-rice ecosystems, the mirid bug *Cyrtorrhinus lividipennis* Reuter feeds on BPH eggs, which it finds by tapping on the stems with their antennae, then sucking the eggs [50]. Further control comes from the hundreds of species of spiders that prey on surviving nymphs and adults [51].

In the absence of these natural enemies, the BPH population can expand exponentially, quickly reaching levels that can cause hopperburn damage that is “comparable to a locust plague in its magnitude of space and intensity” [41] (pp. 286). Adding insult to injury, the BPH also spreads rice grassy stunt and ragged stunt viruses, which can cause serious harm to rice crops even if the adult BPH population is knocked out by heavy spraying [50].

Small patchy hopperburn first appeared in the IRRI farm in 1964, only two years after the onset of field experiments with HYV varieties. By 1971–1972, IRRI was experiencing serious outbreaks. In the mid-1970s, BPH outbreaks were causing major crop damage in Indonesia, southern India and Sri Lanka [45]. IRRI’s response was to undertake a breeding program to develop BPH-resistant rice varieties, crossing the old Peta strand with a Taiwanese variety called “Mudgo” that was distasteful to BPH. Six of these—IR26 and IR30 (released in 1975), and IR24, IR28, IR32 and IR34 (released in 1976), were quickly adopted to replace earlier non-resistant varieties [5]. These strategies were effective for one or two seasons, but by 1977, a new BPH biotype appeared in the Philippines, Indonesia, Vietnam, and Solomon Islands that broke the resistance of the new resistant varieties [41]. IRRI quickly developed a new biotype 2-resistant variety, IR64. But it was becoming clear that new responses were needed [5].

Indonesian scientists also developed and released several new high-quality resistant varieties, by crossing IR36 with local varieties. The BPH problem subsided for several years, and in 1984 Indonesia attained its long-sought self-sufficiency in rice [6]. However, insecticide subsidies continued to increase and ever greater amounts of these chemicals were applied to rice. In 1985–1986, Indonesia witnessed a dramatic and sudden breakdown of resistance to the BPH in all of the IR64-based varieties. In two seasons, an estimated 275,000 hectares of rice were destroyed by BPH, a major threat to the country’s food security [16].

Around this time, researchers found that insecticide use can accelerate the adaptation of pest populations to the resistant varieties [43,52,53]. Natural genetic diversity within planthopper populations is sufficient to provide individuals capable of feeding and reproducing well on the new resistant varieties. Due to their high fecundity and short lifecycle, when insecticides release the BPH from natural enemies, they quickly evolve biotypes that can overcome host plant resistance traits [52].

## 5. The National Integrated Pest Management Program

There is a popular story among IPM aficionados about the origins of integrated pest management in Indonesia. The Indonesian bureaucracy during the New Order period developed a pervasive culture of “*Asal Bapak Senang*”—meaning “as long as Father (one’s superior) is pleased”. Within the Ministry of Agriculture, pest and plant disease infestation records are based on sampling reports of pest observers from the Directorate of Crop Protection. These reports are amalgamated stepwise as they move up through the administrative hierarchy, from sub-district level to the district, province and finally national level. Records of severe pest outbreaks occurring in a jurisdiction are considered a potential embarrassment to one’s supervisor, so estimates of crop damage were routinely underreported, becoming progressively smaller as the reports moved up through the system.

For this reason, the major BPH outbreaks of 1985–1986—especially embarrassing in the wake of the country’s landmark achievement of rice self-sufficiency in 1984—went largely unreported. It was only when farmers from President Suharto’s home village of Kemusuk, near Yogyakarta, made a direct appeal to the President’s brother for help that an independent investigation was launched by the National Development Planning Agency (BAPPENAS), and the extent of the damage became apparent. A politically dangerous situation had been created, and President Suharto was furious [6].

IPM scientists from the national Agricultural University (IPB) in Bogor and from FAO seized this opportunity to act, and in October 1986 actually took several matchboxes of insects and spiders into the President’s office to explain the principles of integrated pest management in rice [54]. President Suharto was so impressed, that he immediately drafted a Presidential Instruction that banned 28 broad-spectrum pesticides in 57 formulations (leaving only ten brands with four active ingredients, all narrow-spectrum insecticides considered effective against BPH), initiated a phasing out of government pesticide subsidies, and mandated integrated pest management (IPM) as the national policy for crop and plant protection. Other features of the instruction included the creation of 1500 new pest observer positions to more than double the number of Directorate of Crop Protection fieldworkers; enforced use of resistant rice varieties; enforced introduction of one secondary food crop after two irrigated rice crops in certain regions; and “crash programs” through “commando posts” (*POSKO*) to deal with pest outbreaks through targeted applications of narrow-spectrum insecticides, if necessary [6].

Initially, IPM was to be promoted through the T&V extension apparatus already in place. The Indonesian government requested permission from the World Bank to divert US$4.19 million remaining from the second phase of the National Agricultural Extension Project (NAEP II) loan to support IPM training. Senior pest observers were trained as “IPM Master Trainers”, and the new Pest Observer recruits and selected extension workers were then given a six-day crash training program. These individuals were to then train contact farmers, in the classic T&V manner. Training guides, flip-charts, slide shows, and thousands of leaflets and pamphlets were hurriedly produced and distributed. Additional funds were expended on travel, honoraria, vehicles, and compensation for farmers attending the courses. The entire budget was expended in seven months, reaching less than ten per cent of the targeted 10,300 farmers. Less than a quarter of those actually entered a rice field during the course of the training program [6]. In post-training assessments, farmers reported that they had not learned much that they could use. Indonesia’s first attempt at implementing integrated pest management in rice proved to be a dismal failure.

### 5.1. A New Approach: Farmer Field Schools

In 1989, USAID provided a grant of US$ 4.7 million to the Indonesian Ministry of Finance and the National Planning Agency (BAPPENAS) to initiate a new two-year pilot IPM Program. Technical assistance was provided by FAO. The decision to house the project in BAPPENAS rather than the Ministry of Agriculture was strategic—it was considered too difficult to devise radical new strategies in an agency so strongly committed to transfer-of-technology T&V extension approaches, and also deeply enmeshed with pesticide company interests. The FAO team established secretariats in Jakarta and Yogyakarta, and worked closely with Directorate of Crop Protection pest observers and selected extension agents in a number of districts in Central and West Java.

The program piloted a new model called Farmer Field School (FFS), which drew from adult non-formal education concepts, based on the assumption that farmers already possess a wealth of experience and knowledge [55]. The other fundamental concept employed was agro-ecological analysis, examining the entire rice paddy agro-ecosystem and relationships between soil, water and weather conditions, rice plants, insect populations and communities, and management activities, rather than focusing just on individual pest problems [56,57,58].

The Farmer Field School takes place over the course of an entire rice growing season, about twelve weeks. The rice field itself becomes a laboratory and a classroom. Often there are two plots: a “non-IPM” plot that is sprayed with pesticides according to Ministry of Agriculture guidelines, and an IPM plot that is managed based on decisions made by the group during their weekly meetings. There are no teachers and students, or trainers and trainees, but rather facilitators and participants (as the program matured, many of the facilitators were themselves FFS farmer-alumni). There are typically about 25 members in an FFS course, who are then divided into groups of five to conduct field observations and analysis. Each session begins with a careful examination of conditions in the fields, observing sample rice hills chosen in a random diagonal transect across each field. The groups make notes of insects, spiders, damage symptoms, weeds and diseases observed on each hill. The stage of plant growth is carefully noted, as are weather and water conditions. Interesting insects and other creatures are captured and placed in small plastic bags. Experiments are set up, to study the effects of plant spacing, water control, varieties, fertilizer and pesticide application, and soil characteristics on plant growth [6,45].

The participants then gather in a nearby home or shed, and prepare drawings of what they observed on large sheets of newsprint. These include the rice plant at its present stage of growth, pests and natural enemies found on the plants, weather, water and soil conditions. Then ensues a lively discussion of the week’s findings, using the “*Apa ini?*” (“What is this?”) principle. Answering a question directly is considered a lost opportunity for learning. Farmers are encouraged to discuss what they have observed, and come to their own conclusions about the status of the crop and possible control measures (or to set up new experiments to find out the answers). One particularly effective activity is the creation of “insect zoos” by placing muslin netting over a rice plant, for farmers to observe predation and parasitism in action, and thereby learn which organisms are pests and which are biological control agents [6].

Special topics are introduced during most sessions, in modules designed to avoid lecturing. These include things like life cycles of rice field inhabitants; parasitism and parasitoids; the effects of pesticides on natural enemies; or the growth of rat populations. Various “ice-breaker” or other socio-dynamic activities enliven the sessions and foster a sense of belonging among the group. These might include competitions and wagers (for instance, whether a newly discovered bug is a predator or pest), with the “losers” having to carry the “winners” around the village on their backs! [59]

Conventional IPM employs the concept of “economic threshold”: *i.e.*, the amount of economic damage that will justify the cost of control. As managers, farmers are expected to weigh expected yield loss (in terms of damage (kg/ha), which they then multiply by price per kg) with estimated pest control costs. For Indonesian rice farmers, this concept seemed overly complex and confusing. In response, the IPM program staff shifted to a concept of “experience threshold”, which develops as farmers learn through experience, and focuses more on the procedure of decision-making [6]. Farmers learn to make crop management decisions based on their personal circumstances and the ecological balance in each paddy.

The four guiding principles of the Indonesian IPM Program reflect this holism and the program’s overall goal of making farmers confident managers and decision-makers, eager for new ideas and information but free from dependence on directives from above:

1. Grow a healthy crop.
2. Observe fields weekly.
3. Conserve natural enemies.
4. Farmers are IPM experts.

Results were immediate and profound: among FFS farmers, insecticide applications reduced from an average of 2.8 sprays per season to less than one, with most farmers not spraying at all. When they did apply an insecticide, IPM farmers could identify a specific pest [45]. Studies showed that IPM farmers had, on average, slightly higher yields, higher overall returns, and lower economic variance than their non-IPM counterparts [16]. But the most impressive change was not in the rice fields *per se*, but in the character of the IPM rice farmers themselves. Farmers and village officials, the lowliest ranked people in the bureaucratic hierarchy and until recently the passive targets and implementers of government directives, were now actively and intelligently discussing their problems, making considered decisions about pest control, producing complex and accurate drawings and diagrams of insects and food webs in their paddy fields, and speaking frankly and openly in front of others—including visiting dignitaries such as the Minister of Agriculture [6].

IPM farmer groups began participating in local Independence Day parades and other festivals, waving banners festooned with integrated pest management slogans, and carrying giant anatomically-correct models of predacious lycosid and linyphiid spiders. From the outset, IPM in Indonesia took on the hallmarks of a movement.

To prepare a core group of trainers for scaling up the program, the program developed a special 15-month Training of Trainers program for 2300 pest observers from the Directorate of Crop Protection. Agricultural extension workers operating under the Agriculture Ministry’s powerful Agency for Agricultural Education and Training and steeped in T&V methodologies, were deemed not particularly suitable candidates for introducing IPM. Ag extension workers have many tasks, among which pest control is a relatively minor role. They are heavily involved—indeed, likely have a vested interest—in input distribution activities, which can conflict with the nature of IPM [6].

The FFS and pest observer training programs were supplemented by a number of research and other activities, including a field laboratory in West Java focusing on the white stemborer (*Scirpophaga innotata* Walker), health impact studies in pesticide-intensive areas in West and Central Java, insect habitat studies, educational and public information materials development, and training evaluation studies. For these research activities, the IPM Secretariat chose to work closely with a few Indonesian and international universities, rather than with the Ministry’s own Research and Development Unit (*Badan Litbang*). Their reasons were the same as for the choice of working with pest observers rather than extension workers: *Badan Litbang* was (and is) highly invested in an input-based agricultural modernization paradigm.

The training evaluation studies were used to constantly refine and improve the FFS model, and devise strategies for “scaling up” [60]. The pesticide studies highlighted the vulnerability of rice farmers to pesticide poisoning and the impact and cost for farmers and the Indonesian people more broadly [61]. Results were shared with farmers during FFS sessions, and participants put dyes in knapsack sprayers to see how much insecticide spray contaminates them when they sprayed their fields.

The IPM program strove to influence national crop management policy and practice. An important opportunity arose in 1990, during a serious outbreak of white stemborer in the key rice producing region of Indramayu, West Java. Indramayu is one of the country’s major rice bowls, with extensive irrigation allowing multiple cropping over a vast area.

Stemborers are particularly injurious pests, and difficult to knock out once a population is established. White stemborer eggs can survive fallow periods in the stubble and stems from the previous harvest [62]. The Ministry’s proposed response to the 1989–1990 outbreak in Indramayu was to reinitiate aerial spraying, a practice that had been discontinued since the 1970s. The IPM Secretariat convinced Ministry officials to hold off while they devised an alternative strategy. Working with local communities and government, the program mobilized hundreds of villagers and school children (who were granted a special school holiday) to descend on rice fields to collect egg masses. This simple action led to a marked reduction in pest pressure, boosting the national standing of the IPM Program considerably [59].

Buoyed by their success, local farmer groups redoubled their efforts to develop strategies to identify more sustainable solutions to this persistent and harmful rice pest. With the assistance of an IPM Field Coordinator, farmers set up a number of experiments to attempt to better understand stemborer biology.

Each year, white stemborer moths would begin appearing soon after the first planting following the annual three-month fallow period. In their first experiment, farmers were shocked to observe that stemborer moths that were collected and sprayed with insecticides still managed to spawn before they died. In another experiment, farmers learned that spraying insecticides directly on egg masses did not prevent healthy larvae from emerging a week later. They then attempted to curb outbreaks by spreading sand coated in insecticide (carbofuran) in seedbeds. Results were variable, depending on the time of application and time of oviposition of stemborer eggs. They had to look for another approach.

The group set up experiments to learn about the origin of the early-season flights. They found live larvae in the stubble of last season’s crops, and so tried burning and flooding the stubble. This, however, did not kill many larvae, and the farmers concluded that physically removing stemborer egg masses was still the most effective method to reduce infestation levels. This is a difficult and time-consuming task. There had to be another way.

The Field Coordinator helped farmers to gain an understanding of aestivation (a form of hibernation during adverse weather conditions), and to design new studies using light traps, in order to more accurately forecast peak flight periods. Stemborer moths have no mouth parts; if they cannot find newly planted rice to lay their eggs within the first week, they die. Using simple rain gauges made from discarded drinking water bottles, the FFS farmers soon learned that the mass flights took place after the first major rainfall of the season. Armed with this knowledge, they were able to organize other farmers in surrounding villages to only plant their nursery beds after peak flights. Once the first flight dies, there are no further generations.

The campaign proved successful, reducing the incidence of white stemborers to less than 5 per cent, compared to 25 per cent in neighboring areas. Through their own experiments, Indramayu farmers were able to devise an effective strategy to solve a problem that has been known by researchers since the 1930s. Coordinated planting of nursery beds has since become the stemborer management strategy for the Indramayu district, a spectacular example of farmer-led agricultural policy [59].

In 1991, the initial two-year program was extended for a third year, with some additional USAID support and some “bridging funds” siphoned from the World Bank National Agricultural Extension (NAEP III) program. The three-year pilot phase ended in 1992. By this time 2300 pest observers had completed the 15-month IPM Training of Trainers course. An additional 6000 agricultural extension workers were also trained in a shorter course. More than 250,000 farmers, in six major rice-producing provinces had participated in FFS courses, corresponding with a 60 per cent reduction in pesticide use in project areas [63].

### 5.2. Scaling Up: IPM Goes Mainstream

Based on the resounding success of the three-year, six province pilot, in 1992 the decision was made to go national. The World Bank provided a loan of $32 million, supplemented by another $7 million from USAID. The main activity was to be the training of approximately 800,000 farmers (30 per cent of these to be women) to develop appropriate, farmer-responsive technology; supplemented by strengthening the regulatory and environmental management of pesticides.

(The World Bank celebrates its support of the popular IPM Program—a search for “Farmer Field School” on the World Bank website conjures links to 745 documents. However, immediately after the first IPM extension debacle in 1986–1987 using left-over Bank loan funds from the National Agricultural Extension Program (NAEP II), the Bank loaned Indonesia another $70 million for T&V extension (NAEP III), more than twice the amount it eventually committed to the National IPM program. Furthermore, during the six-year lifespan of the Indonesian National IPM program, over $350 million of World Bank loan funds were expended globally on pesticide purchase and distribution. A Pesticide Action Network analysis of World Bank projects between 1999 and 2003 indicated that only 9 per cent of projects worldwide complied with the Bank’s own Operational Policy 4.09 on Pest Management, while scores of projects included provisions to “remove bottlenecks” in the procurement and distribution of “agricultural inputs” [64]).

Management of the national IPM program shifted from BAPPENAS to the Ministry of Agriculture. The Indonesian National IPM Program ran from 1993 to 1999. At the same time, the FAO IPM Secretariat in Jakarta became the coordinating center of a 12-country Asia Regional IPM Program.

The program experienced numerous problems related to scaling up. In Indonesia, it took on many of the features of large government projects characteristic of the New Order period, including standardization, emphasis on targets rather than process, lax quality control, leakage (including numerous instances of “ghost” Field Schools), and top-down delivery, as Ministry officials attempted to squeeze and shape the program to fit within pre-existing bureaucratic structures and culture, reverting to T&V command and control models with which they were comfortable [63]. As well, there was constant pressure from both the Ministry of Industry and sections within the Ministry of Agriculture to continue supporting pesticide use in government-led agricultural intensification programs. Meanwhile, the FFS brand became very popular with Indonesian government officials and donors alike (in name if not necessarily in form and spirit), and soon there were “Farmer Field School” projects for artificial insemination and veterinary services, smallholder tree crops, cotton and vegetables [65].

By the time the Indonesian National IPM Program ended in 1999, over a million Indonesian rice farmers had participated in IPM Farmer Field Schools. In combination with somewhat disciplined enforcement of pesticide regulations (the 1986 Presidential Instruction was followed by a 1992 national law on crop management plus a number of other ministerial decrees, until by 2000 the number of banned pesticides formulations reached 120), pesticide misuse in rice was significantly curtailed in Indonesia, and BPH infestation ceased to be a serious problem in the country [63].

At a national workshop held in Yogyakarta in 1999 to conclude a participatory evaluation of the Indonesian IPM Program, FFS alumni formed the National Association of IPM Farmers (IPPHTI, *Ikatan Petani PHT Indonesia*). The FAO Asia Regional IPM Secretariat continued to provide limited support to IPPHTI, primarily for meetings, workshops and organizational development, and to publish a monthly bulletin. FAO also helped secure financial and technical assistance from other funders such as VSO, HIVOS, Diakonia and Norske Foklehjelp—at much reduced levels. The Indonesian Ministry of Agriculture continued to run IPM Field Schools, though the numbers quickly dropped to a trickle.

## 6. Back to the Future: IPM in Indonesia Today

It has now been over fifteen years since the conclusion of the Indonesian National IPM Program in 1999. Indonesia has undergone profound changes during the intervening period. Indonesia was particularly hard hit by the Asian Financial Crisis of 1997–1998. President Suharto resigned in May 1998 after months of political and economic turmoil. The country has since undergone sweeping political and institutional changes, significantly altering the national government’s ability to implement initiatives such as the 1993–1999 National IPM Program.

It is a truism regarding large government development projects that once funding dries up, much of what was constructed begins immediately to degrade. The National IPM Program was no exception to this rule.

Since 2009, after several years of relatively BPH-free rice crops, the insect has returned with a vengeance. This correlates directly with increased insecticide use; Indonesian rice farmers are now spraying more than ever [66,67]. Severe BPH outbreaks have taken place in key rice producing regions of East, Central, and West Java, with both hopperburn and widespread virus infestation occurring. Crop losses in these high-production areas have been significant, approaching or even surpassing the 1985–1986 outbreak that led to the establishment of the national IPM policy and program [67]. The following sections explore the reasons leading to this recurrence.

### 6.1. Reformasi: Democratization and Decentralization Indonesian Style

The end of the National IPM Program coincided with a period of major social and political upheaval in Indonesia. After President Suharto stepped down in May 1998, there ensued a period of rapid political and institutional reconfiguration as Indonesia scrambled to recover from the worst political and economic crisis it had faced since 1965. Democratic reforms introduced by Suharto’s hand-picked successor B.J. Habibie included the repeal of anti-subversion laws, release of political prisoners, freedom of the press, a new law allowing the formation of political parties, Indonesia’s first free legislative elections since 1955, a referendum on the future of East Timor, and a new regional autonomy law that set in motion a rapid and radical devolution of government power and responsibility to regional governments [68,69]. Other changes included significant deregulation of trade and investment [70,71].

With these changes, the context for IPM in Indonesia changed dramatically. Hastily-prepared decentralization laws passed in 1999 fundamentally altered the relationship between Jakarta and regional governments, delegating significant decision-making and implementing powers—and budget resources—to district and municipal governments. Government service delivery initially suffered, as the hierarchical chain of command meticulously constructed over decades of military rule came unraveled [72].

In the agriculture sector, Food and Horticultural Crop Protection Centers and staff came under Provincial government control, while Regional Extension Centers and extension workers became the responsibility of District governments. Interaction and communication between agricultural research and extension services, always weak in Indonesia, virtually ceased [73].

Meanwhile, World Bank and other donor support for agricultural extension in Indonesia and developing countries worldwide had diminished considerably. Emphasis shifted to issues of cost recovery and fiscal (un-)sustainability [40]. Without major donor support, national and local politicians are less likely to invest in extension than in more highly visible projects such as irrigation or road construction. The inherent difficulties in attributing impact make extension an easy target for lower budgetary support [74]. As well, around the world, agricultural research priorities had been shifting for some time from staple foods to export crops [75].

The demise of President Suharto meant that IPM in Indonesia lost a major patron at the highest level of government. Ministry of Agriculture support for IPM, always ambivalent, quickly eroded. The first *Reformasi*-era Minister quietly set about dismantling departmental controls on pesticide distribution and marketing [76].

Decentralization also had the effect of rendering it much more difficult to initiate and coordinate large national programs such as the National IPM Program of 1993–1999. National ministries lost considerable control over sectoral budgets and programs. In principal, ministries’ roles are now largely confined to setting policy and minimum standards for government services, and monitoring of policy implementation by regional governments. While some of the more service delivery orientated Ministries such as Health and Education seriously pursued decentralization agendas, some of the other ministries—notably those engaged in natural resource management such as Forestry, Mining and Agriculture—resisted the process [77]. Structures remained largely unchanged, and these ministries strove to maintain large development budgets—euphemistically intended to support the development agendas of regional governments. These budget allocations generally take the form of *projects* and *programs*. In the realm of agriculture, this translates into *inputs*. In essence, the Ministry of Agriculture’s tactics to retain relevance (and budget share) in the era of regional autonomy often resembles a reincarnation of the BIMAS program; *i.e.*, delivery of packages—of credit, seed, fertilizer, chemicals—to farmer groups. This is inimical to IPM approaches.

As the regional autonomy law came into effect in January 2001, the exuberance over Indonesia’s new political freedom quickly degenerated into tussles over control of natural and fiscal resources. Indonesia’s reforms since 1998 ushered in a new era of “money politics” leading to some distinctly non-democratic outcomes. Despite wide-spread recognition of the phenomenon, there is no clearly delineated meaning for the term “money politics”, nor is it our intention to provide one here. Following McVey on Thailand [78] (pp 16), it is sufficient to note that in post-New Order Indonesia, “money has come to dominate politics at all levels: one must have money to run and one must make money from office too”.

Successfully competing for the office of District Head (*Bupati*) allegedly costs between Rp. 20 to 40 billion (US$ 200–400 thousand) [79]. As public campaign financing is virtually non-existent, the sources of such funds are relatively opaque. Needless to say, politicians accrue considerable debts, which must be repaid. Such repayment often takes the form of *contracts*. Infrastructure projects account for much of this, along with concessions in resource-rich provinces for forest industries, plantations, and other extractive industries. In rice-growing districts, options are generally more limited. Hence, district governments in rice and other food crop producing regions tend to promote input packages (that are then contracted out), similar to those descending from the central government discussed above. This is further compounded by the tendency of governments to pursue populist programs, which again often take the form of subsidies or aid packages.

Another consequence of Indonesia’s decentralization and democratization processes has been the politicization of the bureaucracy [80]. Incoming *Bupati*s award supporters with lucrative portfolios, and sideline those who did not back them in the election [81]. Very few supporters of IPM have flourished in this political milieu. This practice is also particularly corrosive of institutional memory.

### 6.2. The Magic of the Marketplace

Partly as a consequence of the US$ 43 billion rescue package provided by the IMF in the wake of the Asian financial crisis, and with the encouragement of other international donors, since 1998 Indonesia has pursued an aggressive policy of deregulation, privatization and trade liberalization. This initially led to a significant spike in the price of agricultural inputs, placing a significant burden on farmers [82]. One response by the Ministry of Agriculture was to deregulate pesticide imports and distribution. In a 2006 interview, the Minister of Agriculture noted that

*(In 2001) the pesticide business was controlled by a small number of multinational companies and just a few local companies. As a result, the price of pesticides to farmers was relatively high. They were cautious, in fact, reluctant, to use pesticides*.

[83]

He went on to explain that Indonesia could confidently deregulate pesticide imports and sales because Indonesia has already promoted Integrated Pest Management through Farmer Field Schools. Indeed, the country’s achievements had been praised by FAO and WHO. Even though prices had dropped, Indonesian farmers adhered to standards of appropriate timing, precise targeting, and correct dosages.

The interview neglected to mention that government support for IPM Field Schools and IPM more broadly had reduced to a trickle, or that only about two per cent of Indonesia’s estimated 44 million rice farmers and agricultural workers had ever participated in IPM Field Schools.

Local and international companies wasted no time taking advantage of the new (de-)regulations. Within a few years, the number of pesticide brands being sold in Indonesia skyrocketed to over 2700—more than 250 of these for use on paddy [84]. The majority of these are combinations of generic ingredients imported from China; Indonesian companies wishing to market them simply register a trademark with the Ministry of Agriculture’s Pesticide Commission. By 2012, 384 companies were importing more than 50,000 tons of 366 active ingredients to produce the dizzying array of products marketed to farmers and government agencies. Between 2000 and 2012, the total value of pesticide imports to Indonesia increased by a factor of six, from US$ 50 million to just under 300 million [66].

The increased competition touted by the Minister above has given rise to aggressive and sophisticated marketing in the countryside. Villages across Java are festooned with pesticide banners, posters and leaflets. Most villages have several kiosks and shops selling agricultural chemicals. Vendors are offered generous bonuses and prizes for meeting sales targets. They organize seminars, workshops, study tours, gala entertainment events with extravagant door prizes, even one-day pesticide “Farmer Field Schools”. Farmers purchasing Rp. 100,000 (about USD $10) worth of pesticide receive a free tee shirt or cap—thus becoming advertisements themselves. Turn in ten empty pesticide containers for recycling, receive a free tee shirt. Farmers are offered an amount of pesticide free of charge in return for putting up a poster in their field (if the results appear positive). Pesticide companies repair broken backpack sprayers for free. Other promotions include lotteries to win televisions, refrigerators, even Hajj trips to Mecca! [85]

Pesticide companies employ their own field workers, known as “formulators”. These individuals are better educated, provisioned, and remunerated than government extension workers. As well, the companies take advantage of the dire economic situation of most government extension programs and workers, offering incentives to those who successfully promote their products [76]. Agricultural extension workers’ primary responsibility is to collect “Definitive Group Work Plans” from farmer groups; basically a shopping list of inputs requested from government agencies and partner suppliers. This presents a convenient opportunity for close collaboration between extension workers and pesticide distributors.

As a result, more Indonesian farmers are using more pesticides than ever before. If pest problems persist, the general response is to try something more expensive, or to mix different formulations together to create a hopefully more powerful chemical cocktail [86].

Surveys conducted by students from the University of Indonesia and Bogor Agricultural Institute in several rice growing districts in Central Java in 2011 found a dizzying array of pesticide brands being used, comprised mostly of the active ingredients shown in Table 1 [67,87]:

**Table 1:** Partial list of pesticides being used by Rice Farmers in Central Java in 2011.

bisultap	etofenprox
buprofezin	fenobucarb
carbosulfan	fipronil
chlorpyrifos	imidacloprid
cyfluthrin	lambda-cyhalothrin
cypermethrin	metomil
deltamethrin	proproxur
diazinon	thiamethoxam
dimehypo	thiocyclam hydrogen oxalate
dinotefuran	

This list includes at least one active ingredient (the organophosphate diazinon) that was banned for use in rice by the 1986 Presidential Decree that launched Indonesia’s IPM program, plus a number of other broad-spectrum insecticides identified by Bogor Agricultural Institute crop scientists as prone to promote planthopper outbreaks including chlorpyrifos, (organophospate), fipronil (phenylpyrazole), deltamethrin (pyrethroid), and imidacloprid (neonicotinoid) [67]. Some of the other compounds on the above list appear on the Pesticide Commission’s 672-page list of approved ingredients, but are not indicated for use on rice [84]. On the list also is the juvenile retardant buprofezin which is often inappropriately combined with other insecticides. Clearly, the information being provided to Indonesia’s rice farmers has more to do with selling chemicals than with controlling planthoppers.

### 6.3. Masterplanning and Modernizing (Again)

Beginning in about 2004, some seven years and three presidents after the economic and political crises that brought an end to President Suharto’s New Order regime, Indonesia entered a new phase of robust economic growth, driven largely by exports of primary commodities (coal, ore and palm oil) to China, combined with increased domestic consumption. National and many local governments are becoming increasingly confident, and have initiated a number of ambitious modernization programs, under the overarching framework of the *Masterplan for the Acceleration and Expansion of Indonesia Economic Development* (MP3EI). This technology and infrastructure-based Masterplan intends to shift Indonesia from the category of emerging to advanced economy within the next 15 years. The Masterplan sets ambitious targets for each of eight primary programs (agriculture, mining, energy, industrial, marine, tourism, telecommunication, and the development of strategic areas). Food agriculture is one of 22 main economic activities targeted for acceleration and expansion [88].

Within the agriculture sector, the Masterplan is further elaborated in a national strategy known as the Revitalization of Agriculture, Fisheries and Forestry (RPPK), and for rice, a program entitled National Rice Production Increase (P2BN), which targets annual production increases of around six per cent, intended to reach a national rice surplus of ten million tons by 2014.

This discourse of acceleration and expansion, and ambitious production targets, are fodder for the classically trained agronomists and agricultural economists steeped in an input/output-based agricultural modernization paradigm, who dominate Indonesia’s agriculture bureaucracy. Targets justify budgets; budgets purchase and deliver inputs. Then it is simply a matter of getting farmers to do the rest. For the Indonesian Ministry of Agriculture, this marks a return to the halcyon days of the BIMAS program. Tactics—and results—hearken back to the period before the onset of the BPH crisis of the mid-1980s.

The primary vehicle for the delivery of inputs and instructions to farmers is called a Field School, though it bears little resemblance to the original FFS concept established during the National IPM Program. So-called Integrated Crop Management (ICM) Field Schools consist of four two-hour sessions where farmers are introduced to particular techniques and inputs—quite different from the twelve or more half-day meetings comprising the original IPM Field Schools. The *participatory* aspect of ICM Field Schools appears limited to farmers choosing which *packet* they wish to accept. There is no longer any pre-FFS assessment or research by participants, nor—as IPM Field School proponents are quick to point out—no experimentation, experiential learning, laughing or clapping.

The ICM Field School model was developed by the Ministry of Agriculture’s Research and Development Unit (*Badan Litbang—* which had been intentionally circumvented during the original IPM program). Implementation is managed by the powerful Directorate of Cereals in the Directorate General of Food Crops with additional support from the Directorate General of Agricultural Facilities and Provisions. These two agencies have reaped the greatest benefit from the target-based P2BN program, whilst the Directorate of Crop Protection—the institutional home of the IPM Field School—faces a much more difficult task establishing causal links between its work and production increases [89]. Any protests from this group that IPM Field Schools remain fundamentally important to the health and productivity of national agriculture are met with the response that the ICM Field Schools already incorporate IPM principles; that ICM is in effect “*IPM plus*”.

The Directorate of Crop Protection continues to conduct IPM Field Schools—though at a much reduced level than during the National IPM Program. Since 2006 the Directorate has funded about 560 IPM Farmer Field Schools nationally per year, or less than 50 per year in each of the 12 major rice-growing provinces [90]. Compared to the number of ICM Field Schools being supported, this is a mere trickle. In keeping with the production target orientation of the ICM Field Schools, Ministry statistics do not record how many Field Schools were implemented, but rather the number of hectares covered. In 2010, this figure reached 2,393,015 hectares nationally [91], which conservatively equates to about 200,000 Field Schools. To illustrate, in 2012, East Java, Indonesia’s main rice-producing province, received budget support from the Ministry of Agriculture to implement just over 200 IPM Farmer Field Schools. The budget for ICM Field Schools, on the other hand, set a target of between 30 and 40 Field Schools per sub-district, or approximately 20,000 ICM Field Schools in one year [73].

Yet another strategy introduced in recent years to achieve ambitious rice production targets is the introduction and dissemination of hybrid rice. Commercial hybrids are a relatively new innovation in rice technology, promising to break the current rice yield ceiling through the effect called *heterosis*, or hybrid vigor. (Although HYV rice is created through the hybridization of different strands, it is then ‘bred out’ for several generations to achieve a stable genetically fixed, or inbred, variety. Hybrid rice, on the other hand, is the first generation (F1) offspring of crossing two inbred varieties. First generation hybrids express heterosis, often in the form of much higher yields. Second generation (F2) seed produced by the F1 plants is inferior, meaning that farmers need to purchase new F1 seeds every season to achieve the heterosis effect each time).

China has led the way with development and dissemination of hybrid rice seed; presently around 50 per cent of China’s paddy land is planted in hybrid varieties. Most estimates suggest that China’s hybrid rice yields on average 15 to 20 per cent more than inbred HYV varieties, however, the hybrid crops also require much higher dosages of fertilizer and pesticides.

These hybrid strains appear prone to a range of rice pests, including white stemborer (*Scirpophaga innotata* Walker), whiteback planthopper (*Sogatella furcifera* Horvath), rice leaf roller (*Cnaphalocrocis medinalis*), bacterial blight (*Xanthomonas oryzae pv. oryzae*), sheath blight (*Rhizoctonia solani*), downy mildew (*Sclerospora oryzae*, *Sclerophthora macospora*), false smut (*Ustilaginoidea virens*), kernel smut (*Tilletia barclayana*) and various viral diseases (tungro, rice ragged stunt, rice grassy stunt) [92]. Perhaps most significantly, due to a combination of plant physiology, high fertilizer use and overuse of chemical agents to combat the array of pests and diseases above, hybrid rice is extremely susceptible to BPH infestation.

Nonetheless, since 1998, the Asian Development Bank, together with FAO, IRRI and the Asia Pacific Seeds Association (APSA) have been aggressively promoting the introduction of hybrid seeds in Bangladesh, India, Indonesia, the Philippines, Sri Lanka and Vietnam. Initially, seeds were distributed with heavy subsidies to offset the purchase cost (usually about ten times the cost of HYV seed) [92]. In each of these countries, the initial euphoria over yield increases rapidly gave way to farmer resistance, as millers and consumers shied away from purchasing hybrid rice, which they claim is too sticky, too mild, and also has a much lower milled rice to husk ratio [93].

The national government in Indonesia has been distributing hybrid seeds every year since 2004—often through the mechanism of ICM Field Schools. Former President Susilo Bambang Yudhoyono appeared on national television harvesting hybrid rice in several provinces each year between 2007 and 2012. The hybrids have not been popular with farmers, due to high cost, the fact that they cannot keep seed for replanting, susceptibility to BPH and other rice pests, and low consumer demand for the product. Nonetheless, the government continued to promote the technology, targeting an area of over 300,000 hectares by 2014 [94]. After serious BPH outbreaks in 2010–2011 (see below), the Ministry and provincial government of Central Java abandoned these ambitious targets—for the time being, at least.

### 6.4. Return of the Planthopper

Under these conditions, it is hardly surprising that BPH infestations began to manifest in many of Indonesia’s major rice-producing areas. Due to the rather chaotic administrative situation created by the ongoing decentralization process, combined with reluctance to report bad news that still persists in Indonesia despite more than a decade of democratic reforms and press freedom, accurate figures on the scale of the problem are unavailable. Throughout the early years of the 2000s, BPH infestation figures ranged in the low thousands of hectares, but between 2008 and 2011 shot up dramatically. The figures from the Directorate of Crop Protection shown in Table 2 most likely significantly understate the magnitude of the problem [95]: 

**Table 2:** Rice brown planthopper (BPH) Infestation 2008–2013.

Year	Rice Paddy Planted (Java)	Infested (ha)	Totally Destroyed (ha)
2008	5,742,270	24,152	608
2009	6,093,303	47,473	1237
2010	6,358,521	137,768	4602
2011	6,165,079	223,656	36,065
2012	6,185,521	30,174	242
2013	6,467,073	61,630	2763

The sharp decline in 2012 can be explained by two factors. First, in 2011 the government (temporarily, at least) abandoned its policy of promoting hybrid rice, as it became increasingly obvious that the hybrid varieties were especially susceptible to BPH outbreaks, and began distributing BPH-resistant inbred varieties Inpari 11 and 13. Also, 2012 was a year of low rainfall in most of Indonesia, compared to the very wet two previous years [76].

It appears that Indonesia has now come nearly full circle since that day in 1986 when IPM scientists took matchboxes of rice field insects into the office of former president Suharto, which led to the establishment of what has been heralded as one of the most successful national integrated pest management programs in the world [96,97].

The foregoing story provides an important cautionary note not about the sustainability of Integrated Pest Management *per se*, but certainly about the sustainability of government-supported IPM programs, and about the self-sustaining and self-replicating nature of sound agronomic practice grounded in farmer empowerment and mobilization. Despite the considerable success of the Indonesian National IPM Program in the 1980s and 1990s, its accomplishments have withered in the subsequent onslaught of bad policy, worse programs and aggressive and relentless marketing by the producers and traders of agricultural chemicals and seeds.

There remains a committed cadre of IPM die-hards scattered across numerous locations where the IPM Program succeeded two decades ago, but they are growing old. Very few of the 14,000 Pest Observers and IPM Master Trainers who joined the program back in 1990 still work for the government; those who do are nearing retirement age, and a new generation has not been prepped to replace them.

At a recent *ad hoc* meeting of national and international IPM supporters to discuss the ongoing BPH epidemic in Indonesia, one observer mooted the rather pessimistic suggestion that the most effective strategy might be to wait until the problem becomes so serious that the national government has no choice but to act—similar to the situation that spawned the original National IPM Program back in 1986 [76]. If and when that does transpire, an eager army of spiders, water striders, dragonflies, damselflies, beetles, wasps and fungi are waiting in the wings to play their crucial role in supporting Indonesia’s food security and sovereignty. They will be helped along by a much smaller, older, but no less purposeful and dedicated corps of IPM Field School alumni and their dwindling numbers of committed allies still in government service.
